# Quantification of both the area-at-risk and acute myocardial infarct size in ST-segment elevation myocardial infarction using T1-mapping

**DOI:** 10.1186/s12968-017-0370-6

**Published:** 2017-08-01

**Authors:** Heerajnarain Bulluck, Matthew Hammond-Haley, Marianna Fontana, Daniel S. Knight, Alex Sirker, Anna S. Herrey, Charlotte Manisty, Peter Kellman, James C. Moon, Derek J. Hausenloy

**Affiliations:** 1 The Hatter Cardiovascular Institute, Institute of Cardiovascular Science, University College London, London, UK; 2 The National Institute of Health Research University College London Hospitals Biomedical Research Centre, London, UK; 3 Barts Heart Centre, St Bartholomew’s Hospital, London, UK; 4 National Amyloidosis Centre, University College London, Royal Free Hospital, London, UK; 5 National Heart, Lung and Blood Institute, National Institutes of Health, Bethesda, USA; 6 Cardiovascular and Metabolic Disorders Program, Duke-National University of Singapore, Singapore, Singapore; 7 National Heart Research Institute Singapore, National Heart Centre Singapore, Singapore, Singapore; 8 Yong Loo Lin School of Medicine, National University Singapore, Singapore, Singapore

**Keywords:** ST-segment elevation myocardial infarction, Primary percutaneous coronary intervention, Myocardial infarct size, Area-at-risk, T1-mapping, T2-mapping, Cardiovascular magnetic resonance

## Abstract

**Background:**

A comprehensive cardiovascular magnetic resonance (CMR) in reperfused ST-segment myocardial infarction (STEMI) patients can be challenging to perform and can be time-consuming. We aimed to investigate whether native T1-mapping can accurately delineate the edema-based area-at-risk (AAR) and post-contrast T1-mapping and synthetic late gadolinium (LGE) images can quantify MI size at 1.5 T. Conventional LGE imaging and T2-mapping could then be omitted, thereby shortening the scan duration.

**Methods:**

Twenty-eight STEMI patients underwent a CMR scan at 1.5 T, 3 ± 1 days following primary percutaneous coronary intervention. The AAR was quantified using both native T1 and T2-mapping. MI size was quantified using conventional LGE, post-contrast T1-mapping and synthetic magnitude-reconstructed inversion recovery (MagIR) LGE and synthetic phase-sensitive inversion recovery (PSIR) LGE, derived from the post-contrast T1 maps.

**Results:**

Native T1-mapping performed as well as T2-mapping in delineating the AAR (41.6 ± 11.9% of the left ventricle [% LV] versus 41.7 ± 12.2% LV, *P* = 0.72; R^2^ 0.97; ICC 0.986 (0.969–0.993); bias −0.1 ± 4.2% LV). There were excellent correlation and inter-method agreement with no bias, between MI size by conventional LGE, synthetic MagIR LGE (bias 0.2 ± 2.2%LV, *P* = 0.35), synthetic PSIR LGE (bias 0.4 ± 2.2% LV, *P* = 0.060) and post-contrast T1-mapping (bias 0.3 ± 1.8% LV, *P* = 0.10). The mean scan duration was 58 ± 4 min. Not performing T2 mapping (6 ± 1 min) and conventional LGE (10 ± 1 min) would shorten the CMR study by 15–20 min.

**Conclusions:**

T1-mapping can accurately quantify both the edema-based AAR (using native T1 maps) and acute MI size (using post-contrast T1 maps) in STEMI patients without major cardiovascular risk factors. This approach would shorten the duration of a comprehensive CMR study without significantly compromising on data acquisition and would obviate the need to perform T2 maps and LGE imaging.

## Background

Cardiovascular magnetic resonance (CMR) is considered the gold standard imaging modality for quantifying myocardial infarct (MI) size [1]. It can also provide information on the edema-based area-at-risk (AAR) [2, 3], which can be used to calculate myocardial salvage (by subtracting MI size from the AAR) - a more sensitive measure for assessing the efficacy of novel cardioprotection therapies for reducing MI size in reperfused ST-segment elevation myocardial infarction (STEMI) patients [4].

T2-weighted imaging in the first week following primary percutaneous coronary intervention (PPCI) has been used to delineate the AAR in STEMI patients [5], but has recently attracted controversy [6, 7]. T2-mapping [8] has emerged as a more robust technique but has not yet been established as the gold standard for the AAR (therefore referred to as the edema-based AAR throughout the article) and is a topic of ongoing research. Native T1-mapping has been shown to perform well against T2-mapping in a canine model of MI [3], and in clinical patients at 3.0 T [2]. So far, no study has directly compared the performance of T1-mapping against T2-mapping at 1.5 T in these patients.

Conventionally, MI can be identified by late gadolinium enhancement (LGE) with inversion recovery (IR) T1-weighted sequences and manual adjustment of the inversion times (TI) to null the remote normal myocardium. Post-contrast T1-mapping has also shown promise to detect acute MI in a small cohort of predominantly non-reperfused acute MI patients and chronic MI [9, 10]. Refinements of the T1 Modified Look-Locker Inversion recovery (MOLLI) prototype using IR images at different TIs initially designed to improve motion correction [11], can also create “synthetic” IR LGE images, namely magnitude-reconstructed IR (MagIR), phase-sensitive IR (PSIR) outputs. These synthetic read-outs have recently been shown to accurately detect chronic MI size in a small cohort of 17 patients [12]. To date, no studies have assessed the performance of post-contrast T1 mapping and synthetic LGE in quantifying MI size with full left ventricular (LV) coverage in a cohort of reperfused STEMI patients.

A comprehensive CMR study in STEMI can be time-consuming. Therefore, the aims of this study were to investigate, firstly, whether native T1-mapping can accurately delineate the edema-based AAR at 1.5 T (using T2-mapping as the reference); and secondly, whether post-contrast T1-mapping and synthetic IR LGE images can quantify MI size (using conventional LGE as the reference). This may allow one to omit conventional LGE imaging and T2-mapping to quantify MI size and the edema-based AAR, respectively, thereby shortening the duration of the scan.

## Methods

### Study population

Twenty-eight acute STEMI patients reperfused by PPCI were chosen from a cohort reported in previous studies [13–17] based on the availability of full LV coverage of post-contrast T1 mapping and the availability of the synthetic IR output dataset (recruitment period for the whole cohort was August 2013 to July 2014, the option to generate synthetic LGE images from the T1 maps were available from January 2014). The management of STEMI was as per current guidelines. Study exclusion criteria were known previous MI and standard recognized contraindications to CMR. The UK National Research Ethics Service approved this study and all patients provided informed consent. All 28 patients underwent CMR at 3 ± 1 days post-PPCI.

### Imaging acquisition

All CMR scans were performed on a 1.5 Tesla scanner (Magnetom Avanto, Siemens Healthcare, Erlangen, Germany) using a 32-channel phased-array cardiac coil. The protocol included cine CMR images, T2 maps, native T1 maps, T2* maps, early gadolinium enhancement (EGE), conventional LGE and post-contrast T1 maps (All short axis T1/T2 maps, EGE and LGE images were aligned with the short axis cine MR images). Full LV short axis coverage was acquired for all sequences except for T2* maps (basal, mid and apical short axis). The T2* maps and EGE were not analyzed for this specific study but the time taken for each imaging sequence was recorded to the nearest minute to obtain representative duration of a comprehensive CMR study in these patients.

#### T2-mapping (work in progress #448B, Siemens healthcare)

T2 maps were acquired using 3 single-shot images at different T2 preparation times (0 ms, 24 ms, and 55 ms) as previously described [8]. The imaging parameters were: pixel bandwidth 930 Hz/pixel; echo time = 1.1 ms; repetition time = 3xR-R interval; flip angle = 70°; acquisition matrix = 116 × 192; slice thickness = 6 mm; slice gap = 4 mm; voxel size = 2.0 × 2.0 × 6.0 mm; temporal resolution = 190 ms; acceleration factor = 2). Fitting was performed inline to estimate T2 relaxation times and a colored T2 map consisting of pixel-wise T2 values was generated inline following motion correction.

#### Native T1-mapping (work in progress #448B, Siemens healthcare)

T1 maps were acquired using a steady state free precession-based MOLLI prototype. A 5 s(3 s)3 s modified MOLLI sampling protocol was used as previously described [11]. The acquisition parameters were: pixel bandwidth 977 Hz/pixel; echo time = 1.1 ms; flip angle = 35°; matrix = 256 × 144; slice thickness = 6 mm; slice gap = 4 mm; voxel size = 1.5 × 1.5 × 6.0 mm; temporal resolution = 227 ms; acceleration factor = 2). Motion correction and a non-linear least-square curve fitting were performed with the set of images acquired at different inversion times to generate a pixel-wise colored T1 map inline.

#### Late gadolinium enhancement

Conventional Mag-IR LGE imaging was acquired using predominantly a respiratory motion-corrected, free-breathing single shot steady state free precession averaged inversion recovery (MOCO-FB) LGE sequence in 22/28 patients (typical imaging parameters were: bandwidth = 977 Hz/pixel; echo time = 1.48 ms; repetition time = 700-900 ms; flip angle = 50°; acquisition matrix = 144 × 256; slice thickness = 8 mm; slice gap: 2 mm; voxel size = 1.6 × 1.6 × 8.0 mm; temporal resolution = 202 ms; acceleration factor 2) or a standard segmented ‘fast low-angle shot’ (FLASH) two-dimensional IR gradient echo sequence in 6/28 patients (imaging parameters were: bandwidth = 140 Hz/pixel; echo time = 3.17 ms; repetition time = 700-900 ms; flip angle = 21°; acquisition matrix = 125 × 256; slice thickness = 8 mm; slice gap: 2 mm; voxel size = 2.1 × 2.1 × 8.0 mm; temporal resolution = 140 ms; acceleration factor: 2) at 10 min after the injection of 0.1 mmol/kg of Gadoterate meglumine (Gd-DOTA marketed as Dotarem, Guerbet S.A., Paris, France). For both LGE sequences, the inversion times were progressively optimized to null the normal remote myocardium (typical values 360 to 440 ms).

#### Post-contrast T1-mapping (work in progress #448B, Siemens healthcare)

Post-contrast MOLLI T1 maps were obtained in-line using the 4 s(1 s)3 s(1 s)2 s sampling protocol (to improve the accuracy of T1 s in the 200-600 ms range as previously described [11]) 15 min after contrast injection (0.1 mmol/kg of Dotarem) using similar acquisition parameters as for native T1 maps.

**Fig. 1:**
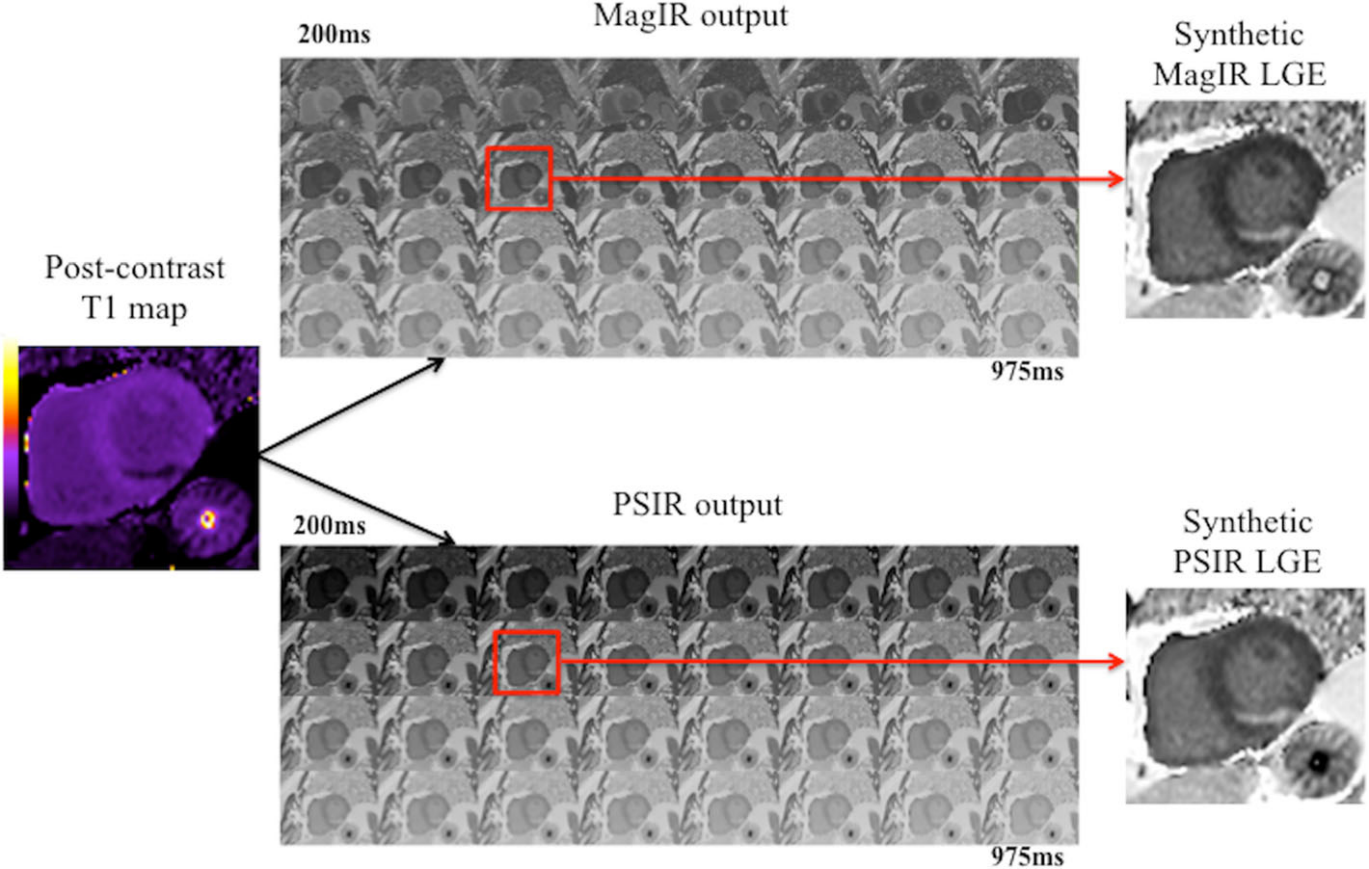
The MagIR and PSIR output from the MOLLI T1-mapping prototype. This is an illustration of the 2 additional outputs (with TI 200 ms to 975 ms) that were obtained from the MOLLI T1-mapping prototype for a patient with an acutely reperfused inferior STEMI. For synthetic MagIR LGE, the image with the optimal TI was manually chosen retrospectively as illustrated by the *red square*. For the synthetic PSIR LGE, the corresponding image from the PSIR output was chosen (*red square*) and was manually windowed if required

Both the native and post-contrast MOLLI T1-mapping automatically generated 2 sets of IR images (MagIR and PSIR) in-line by the scanner for the TI range between 200 to 975 ms (25 ms increments) as illustrated in Fig. 1.

### Imaging analysis

All imaging analysis was performed using CVI42 software (Version 5.1.2, Calgary, Canada).

#### Quantification of MI size

The endocardial and epicardial borders were manually delineated on all the conventional LGE short-axis LV images and were copied to all corresponding images for analysis with minimal manual adjustments. All sequences were subsequently analyzed independent of each other. A reference region of interest (ROI) was drawn in the remote normal myocardium using the automated option available on CVI42 (Circle Cardiovascular Imaging, Calgary, Canada) to minimize variability (with minimal manual adjustment when needed) and this was done for each imaging technique independently. MI size was quantified using a signal intensity threshold of 5 standard deviations (SD) above the normal remote myocardium [12, 18] and expressed as the percentage of the whole LV (%LV). Transmural extent of infarct (TEI) was also quantified and expressed in groups of “no LGE”, “1–25%TEI”, “26–50%TEI”, “51–75% TEI” and “76–100%TEI” as per the 16-segment American Heart Association model.

For the synthetic MagIR LGE, the image with the optimal TI (remote normal myocardium nulled as black) was manually selected and the corresponding matching PSIR image was selected as the representative synthetic PSIR LGE image (Fig. 1). A threshold of 5SD was also used to quantify synthetic MI size.

For the post-contrast T1 maps, we used 2 semi-automated techniques, namely a threshold of 2-SD below the mean remote myocardial post-contrast T1 and the Otsu technique (which identifies the intensity threshold from the signal intensity histogram using the value with minimal intra-class variance between low and high intensities) [19]. These 2 techniques were applied on a slice-by-slice basis. MI size was expressed as: by conventional LGE (reference standard) as MI_Conv_; MagIR synthetic LGE as MI_SynthMagIR_; PSIR synthetic LGE as MI_SynthPSIR_; post-contrast T1 MI size by 2-SD as MI_T1Post2SD_; and by Otsu as MI_T1PostOtsu_.

#### Quantification of the edema-based AAR

The endocardial and epicardial borders were manually traced on the T2 maps and copied to the T1 maps with minimal manual adjustments. In the absence of established quantification techniques, we used manual contouring as reference (%LV) and compared against a threshold of 2-SD above the remote myocardium and the Otsu technique (expressed as AAR_T2Man_, AAR_T22SD_, AAR_T2Otsu_ and AAR_T1Man_, AAR_T12SD_, AAR_T1Otsu_ respectively). The Otsu technique was not applied on maps that were visually normal based on the colored look-up table and with normal wall motion on cine MR images were manually selected as normal.

Areas of hypo-intense core of microvascular obstruction (MVO) were included as part of the MI zone and edema-based AAR. Manual ROIs were drawn in MI zone and the remote myocardium to obtain representative native and post-contrast T1 and T2 values.

### Statistical analysis

SPSS version 22 (International Business Machines Corporation, Chicago, Illinois, US) was used for all statistical analysis. Shapiro-Wilk Test was used to assess for normality and our data was normal distributed. Continuous data were expressed as mean ± SD. Categorical data were reported as frequencies and percentages. Paired student t test was used for edema-based AAR and MI size comparison by different thresholding techniques and by different sequences. T1 and T2 values in the MI zone and remote myocardium were compared with unpaired student t-test. Inter-method correlation was performed using the squared Pearson’s correlation coefficient (R^2^) and inter-method agreement was assessed using intra-class correlation coefficient (ICC) with 95% confidence intervals (95%CI) and Bland-Altman analysis (expressed as bias ±2SD for limits of agreement). All statistical tests were two-tailed, and *P* < 0.05 was considered statistically significant.

## Results

The CMR was performed at 3 ± 1 days post-PPCI. Baseline characteristics are listed in Table 1. The mean age of the STEMI patients was 57 ± 13 years old and 22/28 (79%) were male. The mean MI size (MI_Conv_) in the STEMI patients was 25.1 ± 14.3%LV and the mean edema-based AAR by T2 (AAR_T2Man_) was 41.5 ± 12.0%LV. The myocardial salvage index in this cohort was 0.41 ± 0.28. Out of 448 AHA segments the TEI were as follows: no LGE: 262 (59%); 1–25%TEI: 32 (7%); 26–50%TEI: 60 (13%); 51–75% TEI: 45 (10%); and 76–100%TEI: 49 (11%). 5 patients had TIMI flow grade 3 and 3 patients had TMI flow grade 2 pre-PPCI. The MI size in these 8 patients was significantly smaller than those with TIMI flow 0/1 pre-PPCI (14.0(2.3–25.7)%LV versus 25.0(22.3–34.8)%LV, *P* = 0.04). All patients had areas of hyperenhancement on the LGE areas of high signal on the T1 and T2 maps.

**Table 1:** Patient characteristics and coronary angiographic details

Details	Number
Male	22 (79%)
Age	57 ± 13
Diabetes Mellitus	8 (29%)
Hypertension	8 (29%)
Smoking	10 (36%)
Dyslipidemia	8 (29%)
Chest pain onset to PPCI time (minutes)	216 [138–422]
Infarct artery (%)	
LAD	17 (61%)
RCA	9 (33%)
Cx	2 (7%)
TIMI flow Pre/ Post PPCI (%)	
0	19 (68%)/ 1 (4%)
1	1 (4%)/ 0 (0%)
2	3 (11%)/ 1 (4%)
3	5 (17%)/ 26 (92%)

*LAD* left anterior descending artery, *RCA* right coronary artery, *Cx* circumflex artery, *TIMI* thrombolysis in myocardial infarction, *PPCI* primary percutaneous coronary intervention

**Fig. 2:**
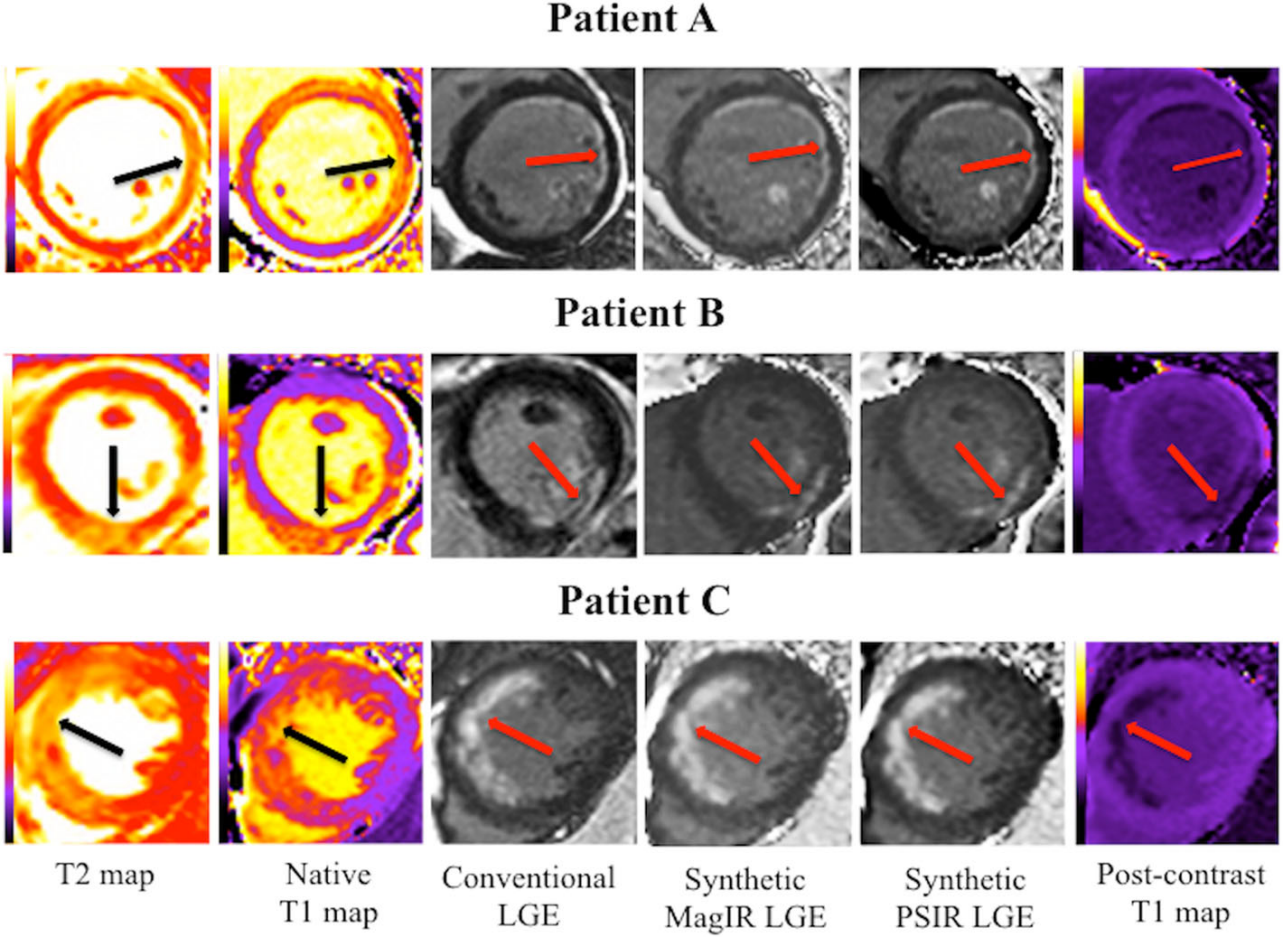
Representative examples of the T2 and native T1 maps showing the edema-based AAR and the corresponding LGE on the conventional, synthetic and post-contrast T1 maps. Patients *A* and *C* suffered from an acute anterior STEMI and Patient *B* suffered from an acute inferior STEMI – all were treated by PPCI. The *black arrows* indicate the territory of the edema-based AAR on the T2 and native T1 maps and the *red arrows* indicate the territory of the subendocardial MI (of different transmural extents) on the conventional and synthetic LGE images and post-contrast T1 maps. All these patients had significant myocardial salvage but the areas of abnormality on the T2 and native T1 maps extended beyond the corresponding MI territory, indicating edema in the salvaged myocardium

Figure 2 illustrates examples from 3 patients for the edema-based AAR by T2-mapping and native T1-mapping and the detection of the acute MI size by conventional LGE, synthetic MagIR LGE and synthetic PSIR LGE, and post-contrast T1 maps.

### T2 and T1 values in MI zone and remote myocardium

T2 in the MI zone was significantly higher than in the remote myocardium (67 ± 7 ms versus 50 ± 3 ms, *P* < 0.0001). Native T1 in the MI zone was also significantly higher than in the remote myocardium (1258 ± 57 ms versus 1041 ± 57 ms, *P* < 0.001). Post-contrast T1 was significantly lower in the MI zone compared to the remote myocardium (441 ± 48 ms versus 625 ± 22 ms, *P* < 0.001).

### Edema-based AAR quantification

The Otsu technique failed in 8% of the maps with extensive MVO and transmural LGE and the edema-based AAR was manually delineated based on the transmural extension of the MI for these slices. There was an excellent correlation (R^2^ 0.99) and agreement (ICC 0.983 and 0.996 respectively) between AAR_T2Man_ and the 2 semi-automated techniques. AAR_T2Otsu_ was similar to AAR_T2Man_ (41.7 ± 12.2%LV versus 41.5 ± 12.1%LV, *P* = 0.38) with no bias (0.2 ± 2.1%). However, AAR_T22SD_ was significantly larger than AAR_T2Man_ (42.2 ± 12.1%LV versus 41.5 ± 12.1%LV *P* = 0.011) with a bias of 1.0 ± 2.6%. (Further details in Table 2).

**Table 2:** Comparison of acute MI size and edema-based AAR quantification by the different techniques

Reference/ % LV	Other Techniques/ % LV	*P* value	R^2^	ICC (95% CI)	Bias ± 2SD/ % LV
Acute MI size					
MI_Conv_ 25.1 ± 14.3	MI_SynthMagIR_ 24.9 ± 13.8	0.35	0.99	0.996 (0.992–0.998)	0.2 ± 2.2 (−2.0–2.4)
	MI_SynthPSIR_ 24.7 ± 13.9	0.060	0.99	0.996 (0.992–0.998)	0.4 ± 2.2 (−1.8–2.6)
	MI_T1Post2SD_ 24.9 ± 13.8	0.10	0.99	0.996 (0.991–0.998)	0.3 ± 1.8 (−1.5–2.1)
	MI_T1PostOtsu_ 23.0 ± 12.9	0.001*	0.96	0.964 (0.859–0.987)	2.2 ± 6.0 (−3.8–8.2)
T2-mapping					
AAR_T2Man_ 41.5 ± 12.1	AAR_T2Otsu_ 41.7 ± 12.2	0.38	0.99	0.983 (0.964–0.992)	0.2 ± 2.1 (−1.9–2.3)
	AAR_T22SD_ 42.2 ± 12.1	0.011*	0.99	0.993 (0.980–0.997)	1.0 ± 2.6 (−1.6–3.6)
T1-mapping					
AAR_T1Man_ 41.0 ± 12.0	AAR_T1Otsu_ 41.6 ± 11.9	0.17	0.99	0.995 (0.990–0.998)	−0.6 ± 4.2 (−4.2–3.6)
	AAR_T12SD_ 42.1 ± 12.2	0.005*	0.96	0.983 (0.949–0.993)	−1.0 ± 3.9 (−4.9–2.9)
T2-mapping versus T1-mapping					
AAR_T2Man_ 41.5 ± 12.1	AAR_T1Man_ 41.0 ± 12.0	0.11	0.99	0.989 (0.977–0.995)	0.5 ± 3.4 (−2.9–3.9)
AAR_T2Otsu_ 41.7 ± 12.2	AAR_T1Otsu_ 41.6 ± 11.9	0.72	0.97	0.986 (0.969–0.993)	−0.1 ± 4.2 (−4.3–4.1)
AAR_T22SD_ 42.2 ± 12.1	AAR_T12SD_ 42.1 ± 12.2	0.86	0.97	0.985 (0.967–0.993)	0.1 ± 4.3 (−4.2–4.4)

*%LV* percentage of the left ventricle, *ICC* intraclass correlation coefficient, *SD* standard deviation, *MI* myocardial infarction, *Conv* conventional, *SynthMagIR* synthetic magnitude reconstruction inversion recovery, *SynthPSIR* synthetic phase sensitive inversion recovery

The same pattern was observed for T1-mapping. The AAR_T1Otsu_ was similar to AAR_T1Manual_ (41.6 ± 11.9%LV versus 41.0 ± 12.0%LV, *P* = 0.17) with no bias (−0.6 ± 4.2%), and AAR_T12SD_ was significantly higher than AAR_T1Manual_ (42.1 ± 12.2%LV versus 41.0 ± 12.0%LV, *P* = 0.005) with a small bias of 1.0 ± 3.9%. (Further details in Table 2).

**Fig. 3:**
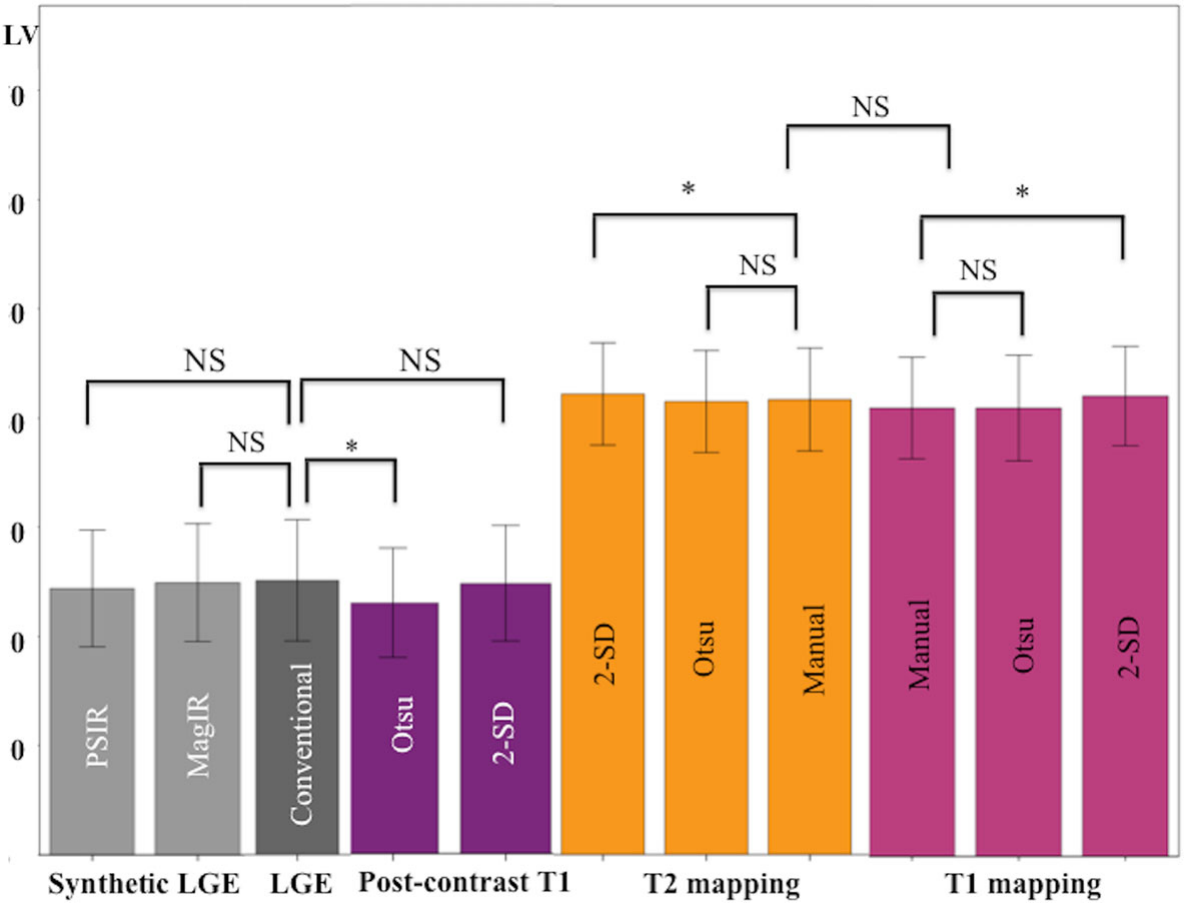
Comparison of MI size and edema-based AAR quantified by the different techniques. This figure illustrates MI size by the conventional LGE, synthetic LGE and post-contrast T1-mapping and edema-based AAR by T2 and T1-mapping. NS denotes no statistical difference and * denote *P* < 0.05. Data presented as mean ± 95% CI

**Fig. 4:**
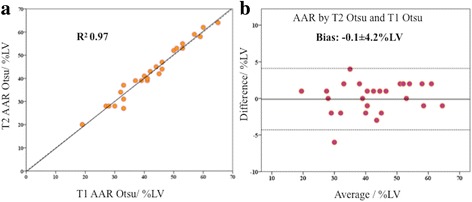
Comparison of the edema-based AAR by T2 and native T1-mapping. There was an excellent correlation (**a**) and minimal bias (**b**) in edema-based AAR delineated by T2-mapping versus native T1-mapping

Native T1-mapping performed as well as T2-mapping in delineating the edema-based AAR (AAR_T1Otsu_ 41.6 ± 11.9%LV versus AAR_T2Otsu_ 41.7 ± 12.2%LV, *P* = 0.72; R^2^ 0.97; ICC 0.986(0.969–0.993); bias −0.1 ± 4.2%LV). Table 2 and Fig. 3 provide further details of the comparison between T2 and native T1-mapping for the quantification of the edema-based AAR using the different techniques. Figure 4 illustrates the comparison between AAR_T2Otsu_ and AAR_T1Otsu_.

### Acute MI size quantification

**Fig. 5:**
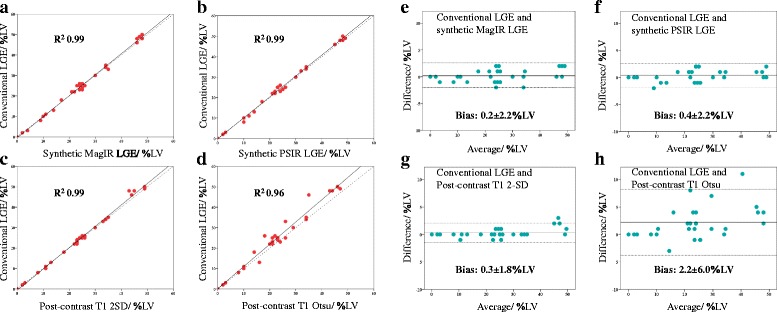
Comparison of MI size by different methods against the reference standard. These are the correlations (**a**-**d**) and Bland-Altman analyses (**e**-**h**) of MI size by conventional LGE against each of the 4 other techniques (synthetic MagIR LGE, synthetic PSIR LGE, post-contrast T1 2-SD and post-contrast T1 Otsu)

There was an excellent correlation (R^2^ 0.96 to 0.99) and inter-method agreement (ICC 0.964 to 0.996) between conventional LGE and the other techniques. There was minimal bias between MI_Conv_ and MI_SynthMagIR_ (bias: 0.2 ± 2.2%LV, *P* = 0.35), MI_Conv_ and MI_SynthPSIR_ (bias: 0.4 ± 2.2%LV, *P* = 0.060) and MI_Conv_ and MI_T1Post2SD_ (bias: 0.3 ± 1.8%LV, *P* = 0.10), with the latter having the narrowest limits of agreement. However, MI_T1PostOtsu_ underestimated MI size when compared to MI_Conv_ (bias −2.2 ± 6.0%LV, *P* = 0.001). Figure 3 shows the comparison of the various techniques used for MI size. Figure 5 illustrates the correlations and Bland-Altman analysis of MI_Conv_ against the 4 other techniques and further details are summarized in Table 2.

### CMR scan duration

**Fig. 6:**
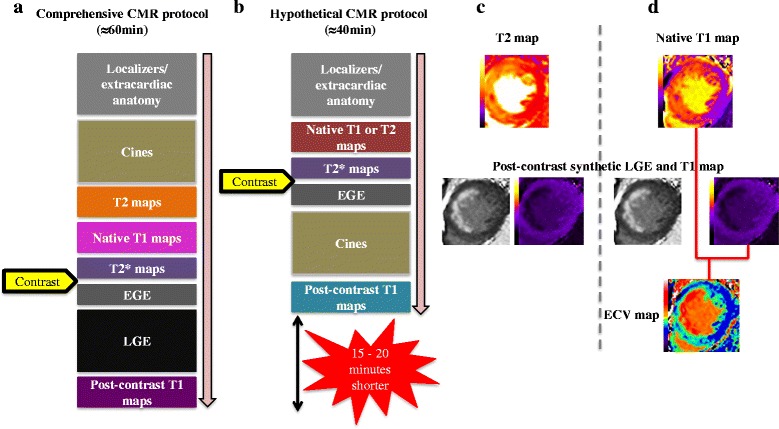
**a**-**d** Duration of the CMR scan. **a** and **b** illustrate the duration for a comprehensive STEMI CMR acquisition protocol providing data on edema-based AAR, intramyocardial hemorrhage, microvascular obstruction, MI size and ECV and on average takes around one hour to acquire. Based on the results from this study, T1-mapping may allow T2-mapping and LGE to be omitted thereby shortening the scanning time by 15–20 min. **c** and **d** illustrates the options of either doing T2-mapping and post-contrast T1-mapping or native T1 mapping and post-contrast T1 mapping depending on the research question

The average time for the complete STEMI CMR scan was 58 ± 4 min (including natural breaks between sequences). The average scanning time for the T2 and T1 maps were 6 ± 1 min each and for LGE it was 10 ± 1 min. Figure 6 illustrates the hypothetical 15–20 min reduction in scanning time obtained from potentially omitting T2-mapping and LGE images to determine the edema-based AAR and MI size, respectively.

## Discussion

The main findings from our study are as follows: firstly, the edema-based AAR can be accurately quantified using native T1-mapping when compared to T2-mapping at 1.5 T; secondly, the acute MI size can be accurately quantified from the post-contrast T1 maps (2-SD) and the synthetic LGE images when compared to conventional LGE imaging; and finally, using T1-mapping to quantify the edema-based AAR and MI size may allow one to omit T2 mapping and LGE image, thereby shortening the scanning time by 15–20 min.

We have previously shown that native T1-mapping can accurately quantify the edema-based AAR at 3 T (analyzed by the Otsu technique) when compared to T2-mapping [2]. Other groups [20, 21] have shown that the Otsu technique also performed well on T2-weighted STIR images to detect the edema-based AAR. However, when using the Otsu technique, manual input is required when no edema is present (using visual assessment of the colored maps and cine MR images as reference), and when extensive MVO is present on the maps. Langhans et al. [22] also demonstrated that native T1-mapping and T2-mapping quantified the AAR when compared to SPECT at 1.5 T, although a direct comparison of T1 against T2 was not performed in that study. We have now shown that native T1-mapping can also accurately delineate the edema-based AAR when compared to T2-mapping at 1.5 T in the clinical setting and that the Otsu technique performed best against manual contouring. The limits of agreement between T2 and T1-mapping were ±4.2%LV, but narrower than our previous study at 3 T (±5.1%LV). T2 mapping is sensitive to the water content and therefore performs well in the context of edema during acute MI. Inversion recovery-based T1 mapping is influenced by T2 due to the use of a steady-state free precession readout leading to a T2 dependent error in the estimation of T1 [11]. Furthermore, a T2-dependent error also occurs due to the imperfect inversion efficiency of the adiabatic radiofrequency preparation. These factors result in the T1 maps being T2-weighting and sensitive to edema. Both T1 and T2 mapping are also equally affected by MVO and IMH [17]. Therefore, it is not surprising that they performed equally well to quantify the AAR. The edema-based AAR by T2 mapping has been shown to be dynamic within the first week of a STEMI [7, 23]. Whether the AAR by T1-mapping will follow the same pattern or would be more stable within the first week of a STEMI is not known and warrants further investigations.

Messroghli et al. [9] have previously shown that post-contrast T1 mapping could detect both acute and chronic MI in a cohort of 24 patients but not all patients underwent coronary angiography prior to CMR and only 21% showed evidence of reperfusion. Bauner et al. [10] have subsequently shown that post-contrast T1 could accurately detect chronic MI in 26 patients. Synthetic LGE has also been shown to accurately quantify chronic MI size in a small cohort of patients [12], but in this study, only one short-axis LV image was analyzed. In our study, we performed full LV coverage and we have shown that post-contrast T1 maps can accurately quantify acute MI size (using the semi-automated 2-SD threshold) when compared to the reference standard in a cohort of reperfused STEMI patients. Using the SD technique for quantification can be variable depending on where the ROI is drawn. To minimize the error associated with this, we have used the automated ROI delineation option with minimal manual adjustment when required. Furthermore, we have also shown that the additional sets of IR images that can be easily obtained from the post-contrast MOLLI T1 maps can be used to obtain synthetic LGE images (both MagIR and PSIR) and can accurately quantify acute MI size. PSIR LGE is T1 weighted with retrospective nulling of the remote myocardium thus being insensitive to the inversion time used for acquisition [24]. With the PSIR technique, the tissue with the longest TI nulls first, irrespective of the TI used (post gadolinium, the remote myocardium has the longest TI) and this sequence has previously been shown to improve MI detection more accurately by LGE compared to conventional MagIR LGE images [25]. Therefore, the synthetic PSIR output could complement post-contrast T1 maps for MI size quantification without the need for conventional LGE images, which requires the user to manually adjust the TI. Post-contrast T1 maps have previously been used as the truth standard for LGE imaging in the setting of cardiac amyloidosis, where nulling of the myocardium is challenging [26]. Therefore, post-contrast T1 mapping with the synthetic LGE output may be useful to identify the pattern of scarring in other pathologies with focal fibrosis such as hypertrophic cardiomyopathy, sarcoidosis and myocarditis and warrants further investigation.

A comprehensive CMR study in a reperfused acute STEMI patient requiring data on the edema-based AAR, intramyocardial hemorrhage (IMH), MVO, MI size and ECV, would take on average, an hour to perform. Based on the results from this study, T2-mapping and conventional LGE could be omitted - this would substantially shorten the scanning time by 15–20 min (see Fig. 6a and b) without compromising data acquisition. This approach would significantly improve patient workflow, make the CMR scan more tolerable to acute STEMI patients, and may help minimize patient dropout in future clinical studies. In our center this could be achieved by omitting T1 or T2 mapping and conventional LGE and performing the short axis cine MR images post-contrast instead (that could also provide another method for the AAR) [27] as illustrated in Fig. 6. Native T1-mapping (for AAR) and post-contrast T1 mapping (for LGE) approach has added value in that the ECV can be derived (Fig. 6d), and this may also be accelerated if the ECV maps are generated inline [28]. Alternatively, If only data on edema-based AAR and MI are required, the T1-mapping could be replaced by T2-mapping (Fig. 6c). The abbreviated protocol could be further shortened in patients who cannot tolerate long scans by omitting the T2* maps (for IMH) and EGE images (for early MVO) and could bring down the scan time to around 30 min. We recently showed that the presence of a hypo-intense core on the T1 maps or T2 maps can detect IMH well and with good sensitivity (85–88%) and specificity (85%) and therefore could be used to detect IMH in situations when T2* mapping cannot be performed or is not available [17].

The appearance of the synthetic MagIR and PSIR is noisy in regions such as air or lungs where the signal to noise ratio is low and the T1 estimates are noisy. This appearance of increased noise in these non-tissue regions is similar to the appearance of PSIR LGE images that results due to correction of surface coil intensities. Surface coil correction is also known as normalization and is generally accompanied by noise amplification in non-tissue regions. Various schemes are often employed to improve the appearance by softening the normalization in these regions. However, these methods may result in some degree of error in tissue regions. This issue, albeit mostly cosmetic, was not addressed in this article and represents a potential impediment to acceptance by clinicians.

### Limitations of study

The conventional LGE scan (acquired 10–15 min post contrast) and post-contrast-T1 maps (acquired 15–20 min post contrast) were acquired 5 min apart. But despite this, there was no difference in MI size. The TI range for the IR images generated in-line from the T1 maps was quite wide (200 ms to 975 ms and at 25 ms increments). But off-line post-processing was not necessary and synthetic MagIR LGE images (Fig. 2) were interpretable and performed as well as conventional LGE for MI size quantification. Our sample size was small but still larger than previously published studies on the edema-based AAR by T1 and T2 mapping [2] and synthetic LGE [12]. We used the edema as a surrogate marker for the AAR, although controversy exists over whether it exists at all [6] or there is a bimodal or unimodal pattern of edema in the first week of an MI [7, 23]. We used 2 different LGE read-outs (FLASH LGE in 22 patients and MOCO-FB LGE in 6 patients) for MI size quantification but both these sequences have been shown to perform equally well for MI size quantification [29]. For some centers not performing such a comprehensive study for STEMI, their scanning time may not be shortened significantly as they would still need to wait 15 min to perform the post-contrast T1 maps. However, they would have the benefit of having 3 outputs to quantify MI size by simply performing post-contrast T1 mapping instead of conventional LGE without having to manually null the remote myocardium. There was a period of 3 months overlap between recruitment of patients in this study and our previous study on T1/T2 at 3 T [2] (part of a positron emission tomography (PET)/MR study) and there may have been an element of selection bias with those >45 years of age and without a history of diabetes being more likely recruited to the latter studies [2, 30]. We have previously shown that the T1 values in the remote myocardium is higher that normal in the acute phase of a STEMI when compared to controls and this might have affected the n-SD technique where an ROI in the remote myocardium is used as reference. There is a regional variation in T1 [31] and T2 [32] values from base to apex, partly contributed by thinner walls and more partial volume effects towards the apex. However, we did not use one cut-off value as threshold for the whole LV but analysis was done on a per-slice basis (ROIs for the 2SD technique were drawn on each slice and the Otsu technique was applied on each slice) to obtain the extent of hyperenhancement. Only around a third of our cohort had the conventional cardiovascular risk factors and therefore these findings apply to this select cohort of STEMI patients only and further studies are required to assess whether synthetic LGE derived from post-contrast T1 maps could be useful in a wider range of patients with STEMI and other pathologies.

## Conclusions

T1-mapping can accurately quantify both the edema-based AAR (using native T1 maps) and acute MI size (using post-contrast T1 maps) in this select group of low-risk STEMI patients, obviating the need to perform T2 maps and LGE imaging, thereby shortening the duration of a comprehensive CMR study without significantly compromising on data acquisition. This approach would make the CMR scan more tolerable to acute STEMI patients.

## Abbreviations

AAR: area-at-risk
CMR: cardiovascular magnetic resonance
EGE: early gadolinium enhancement
FLASH: fast low-angle shot
ICC: intra-class correlation coefficient
IMH: intramyocardial hemorrhage
LGE: late gadolinium enhancement
LV: left ventricular
MagIR: magnitude-reconstructed inversion recovery
MI: myocardial infarct
MOCO-FB: motion-corrected, free-breathing
MOLLI: modified Look Locker Inversion recovery
MVO: microvascular obstruction
PPCI: primary percutaneous coronary intervention
PSIR: phase-sensitive inversion recovery
ROI: region of interest
SD: standard deviation
STEMI: ST-elevation myocardial infarction
TEI: transmural extent of infarct
TI: inversion time
